# Regulatory T Cell Mimicry by a Subset of Mesenchymal GBM Stem Cells Suppresses CD4 and CD8 Cells

**DOI:** 10.3390/cells14080592

**Published:** 2025-04-14

**Authors:** Amanda L. Johnson, Harmon S. Khela, Jack Korleski, Sophie Sall, Yunqing Li, Weiqiang Zhou, Karen Smith-Connor, John Laterra, Hernando Lopez-Bertoni

**Affiliations:** 1 Hugo W. Moser Research Institute at Kennedy Krieger, Baltimore, MD 21205, USAsalls@kennedykrieger.org (S.S.); liyu@kennedykrieger.org (Y.L.); connorka@kennedykrieger.org (K.S.-C.); 2 Department of Neurology, Johns Hopkins University School of Medicine, Baltimore, MD 21205, USA; 3 Department of Internal Medicine, Mayo Clinic, Rochester, MN 55905, USA; 4 Department of Biostatistics, Johns Hopkins Bloomberg School of Public Health, Baltimore, MD 21205, USA; wzhou14@jhu.edu; 5 Department of Oncology, Johns Hopkins University School of Medicine, Baltimore, MD 21205, USA; 6 Department of Neuroscience, Johns Hopkins University School of Medicine, Baltimore, MD 21205, USA; 7 Sidney Kimmel Comprehensive Cancer Center at Johns Hopkins, Baltimore, MD 21205, USA

**Keywords:** TGFBR2, GBM, GSC, immunosuppression

## Abstract

Attempts to activate an anti-tumor immune response in glioblastoma (GBM) have been met with many challenges due to its inherently immunosuppressive tumor microenvironment. The degree and mechanisms by which molecularly and phenotypically diverse tumor-propagating glioma stem cells (GSCs) contribute to this state are poorly defined. In this study, our multifaceted approach combining bioinformatics analyses of clinical and experimental datasets, single-cell sequencing, and the molecular and pharmacologic manipulation of patient-derived cells identified GSCs expressing immunosuppressive effectors mimicking regulatory T cells (Tregs). We showed that this immunosuppressive Treg-like (ITL) GSC state is specific to the mesenchymal GSC subset and is associated with and driven specifically by TGFβ type II receptor (TGFBR2) in contrast to TGFBR1. Transgenic TGFBR2 expression in patient-derived GBM neurospheres promoted a mesenchymal transition and induced a six-gene ITL signature consisting of *CD274* (PD-L1), *NT5E* (CD73), *ENTPD1* (CD39), *LGALS1* (galectin-1), *PDCD1LG2* (PD-L2), and *TGFB1*. This TGFBR2-driven ITL signature was identified in clinical GBM specimens, patient-derived GSCs, and systemic mesenchymal malignancies. TGFBR2^high^ GSCs inhibited CD4+ and CD8+ T cell viability and their capacity to kill GBM cells, effects reversed by pharmacologic and shRNA-based TGFBR2 inhibition. Collectively, our data identify an immunosuppressive GSC state that is TGFBR2-dependent and susceptible to TGFBR2-targeted therapeutics.

## 1. Introduction

The GBM tumor microenvironment (TME) is defined by the low infiltration of anti-tumor immune cells, the high prevalence of T cell exhaustion, and relatively high numbers of suppressive, pro-tumor immune cell infiltrates [1,2]. Tumor cell-intrinsic mechanisms leading to immunosuppressive TMEs are increasingly recognized as barriers to anti-tumor immunity and immunotherapy. These escape mechanisms are utilized by glioma stem-like cells (GSCs) to avoid recognition by the immune system and allow for continued tumor growth [3,4,5]. GSCs can regulate the immune TME by producing factors that recruit immunosuppressive cells and inhibit cytotoxic T cells [5].

Subsets of GSCs possess unique phenotypic and immune-modulatory traits [6]. These glioma stem cells (GSCs) arise through the reprogramming of more differentiated tumor cells induced by specific transcription factors, known as the Yamanaka factors (e.g., Oct4, Sox2, c-Myc, Klf4) [7,8]. Our lab previously described the functions of Oct4 and Sox2 in driving the de-differentiation of GBM cells into GSCs, highlighting the ability of Oct4/Sox2 to enhance tumorigenicity and tumor cell-mediated immune suppression [5,9]. Emerging evidence indicates that tumor cell phenotypic transitions dynamically contribute to establishing and maintaining the immunosuppressive TME in GBM [10,11] and recent observations suggest that GBM dynamically adapts to different modes of immunotherapy by remodeling the tumor cell subtype composition [12], with mesenchymal transitions being of particular importance [10,13]. Which cell fate-determining events contribute to immune evasion in GBM and what aspects of these tumor cell transitions are amenable to therapeutic intervention remain unknown.

One critical mediator of immune regulation in the TME is TGFβ, a cytokine secreted by various cell types with context-dependent immune-regulatory functions [14]. In GBM, TGFβ suppresses anti-tumor immune cells (e.g., T cells, dendritic cells) and promotes pro-tumor immune cells (e.g., tumor-associated macrophages, microglia, and regulatory T cells) [15]. TGFβ signaling is also a well-established driver of stem-like traits and mesenchymal transition in GBM and other malignancies [15]. Canonical TGFβ signaling is initiated by ligand binding to the type II receptor (TGFBR2) that phosphorylates the type I receptor (TGFBR1) prior to the downstream activation of the transcriptional regulators Smad2 and Smad3 by phosphorylation. TGFβ receptors are serine–threonine kinases with a variety of potential interacting proteins and downstream signaling effectors that can signal independently of each other. The distinct roles of TGFBR1 and TGFBR2 in cancer, especially as they pertain to immune regulation in GBM, remain undetermined.

The goal of this study is to further understand how stem cell-driving events contribute to the cell-intrinsic immunosuppressive phenotype of GBM cells. By combining the molecular manipulation of GBM cells, single-cell sequencing, computational analyses, and tumor-immune cell co-culture systems, we identify a novel TGFBR2-driven phenotype in GSCs with molecular parallels to regulatory T cell (Treg) fate induction. We show that mesenchymal-like GBM neurospheres enriched with GSCs (mGSCs) express high levels of TGFBR2 and are capable of repressing CD4+ and CD8+ T cell viability and function in vitro. Moreover, TGFBR2 inhibition reverses this immunosuppressive tumor cell phenotype by reducing CD8+ T cell exhaustion and enhancing CD4+ and CD8+ T cell-mediated tumor cell killing. These findings identify a novel mechanism of GSC immunosuppression and highlight the potential applicability of anti-TGFBR2-specific therapeutics for augmenting immunotherapy in GBM.

## 2. Materials and Methods

### 2.1. Human Cell Culture

All GSCs used in this study were derived from newly diagnosed GBMs (IDH-wildtype) and cultured in serum-free conditions using Stemline Neural Stem Cell Expansion Medium (Sigma-Aldrich, St. Louis, MO, USA) supplemented with 20 ng/mL epidermal growth factor and 10 ng/mL fibroblast growth factor. The classical-like patient-derived neurosphere lines, GBM1A and GBM1B, were originally derived and characterized by Vescovi and colleagues [16]. The mesenchymal-like patient-derived GBM xenograft cell line, Mayo39, was obtained from the Mayo Clinic (Rochester, MN, USA) [17] and enriched with stem-like cells by culturing in Stemline Neural Stem Cell media prior to use in experiments. Low-passage patient-derived mesenchymal GSCs, M1123, were a kind gift from Dr. Nakano at The Ohio State University [18]. HEK293FT cells were obtained from the ATCC and grown in Dulbecco’s modified Eagle medium (DMEM) supplemented with 10% FBS (fetal bovine serum, Thermo Fisher, Waltham, MA, USA). Prior to experimentation, all cells were tested for mycoplasma contamination and authenticated via STR profiling.

### 2.2. Lentivirus Generation and Cell Transduction

The transgenic cell lines used in this study were generated with the second-generation lentiviral system according to Addgene protocols, using the psPAX2 packaging plasmid and pMD2.G envelope plasmid (Addgene, Watertown, MA, USA). The lentiviral packaging/envelope plasmids and the transgene vector (Supplementary Table S1) were co-transfected into HEK293FT cells using the Lipofectamine 3000 kit (Thermo Fisher) according to the manufacturer’s recommendations. The next morning, sodium butyrate (Cayman Chemical, Ann Arbor, MI, USA) was added to transfected cells at a final concentration of 10 mM to enhance the viral titer. After 48 h, lentiviral particles in the supernatant were concentrated using Lenti-X concentrator solution (Takara Bio, San Jose, CA, USA) and resuspended in 1 mL PBS to transduce cells. GSCs were infected overnight with the lentivirus particles plus 1 ug/mL polybrene. The next morning, cells were replated in fresh neurosphere media.

### 2.3. Western Blot Analysis

To measure protein expression, cells were lysed in RIPA buffer (Sigma-Aldrich) plus protease inhibitors (Sigma-Aldrich #P8340) and phosphatase inhibitors (Sigma-Aldrich #P5726) for 30 min on ice. Protein was purified by centrifugation and quantified by the Bradford protein assay. Equal quantities of protein were loaded per sample (40–80 μg) and resolved on a NOVEX 4–12% or 4–20% Tris-glycine gradient gel (Thermo Fisher) using the Thermo Fisher Mini Gel Tank system. The protein was then transferred onto an Amersham Protran nitrocellulose membrane (GE Healthcare, Chicago, IL, USA) using the Bio-Rad Mini Protean 3 Cell system. The membrane was blocked for 1 h in Li-COR Intercept blocking buffer before primary antibodies (Supplementary Table S2) were added and incubated overnight at 4 °C. Membranes were then washed and incubated with infrared-labeled secondary antibodies (Li-COR Biosciences, Lincoln, NE, USA) prior to quantification using the Odyssey CLx Infrared Imager (Li-COR Biosciences). Densitometry analysis was performed using Image Studio 6.0 software from Li-COR imaging systems. Protein expression was normalized to the loading control (e.g., GAPDH).

### 2.4. qRT-PCR Analysis

To measure gene expression, total RNA was extracted from cells using the RNeasy Mini Kit (Qiagen, Germantown, MD, USA) and converted to cDNA by reverse-transcribing 500 ng−1 μg of RNA using MuLV Reverse Transcriptase and Oligo (dT) primers (Applied Biosystems, Waltham, MA, USA). Expression was measured using the Power SYBR Green PCR kit (Applied Biosystems) and quantified using a Bio-Rad CFX96 Real-Time Detection System and accompanying Bio-Rad CFX manager v3.1 software. Samples were produced in triplicate and the signal was normalized to 18S RNA. Primer sequences are provided in Supplementary Table S3.

### 2.5. Cell Viability and Cell Death Assays

To quantify cell viability and cell death, cells were split evenly and incubated at room temperature (RT) in either Cell Titer Glo (Promega, Madison, WI, USA) or Caspase 3/7 Glo (Promega) at a 1:1 (*v*/*v*) ratio to cell media. After 30 min, luminescence was measured using the SpectraMax M5 Multimode Plate Reader (Molecular Devices, San Jose, CA, USA) and quantified using SoftMax Pro 7 software. Cell viability was calculated as the ratio of the Cell Titer Glo to Caspase 3/7 Glo signal, whereas cell death was represented by the inverse value.

### 2.6. Flow Cytometry and FACS

To quantify cell proportions, GSCs were dissociated into single cells and incubated with the appropriate fluorescently labeled antibodies (Supplementary Table S4) following the manufacturer’s recommendations (Miltenyi Biotec, Gaithersburg, MD, USA). Quantification was performed using the Muse^®^ Cell Analyzer (Sigma-Aldrich) and gated for cell size and fluorescence signals. To sort the GSCs into high- and low-CD44 fractions, the GSCs were processed in the same way as above and sorted using the MoFlo Astrios EQ cell sorter (Beckman Coulter, Brea, CA, USA).

### 2.7. RNA Sequencing

RNA-Seq libraries were constructed from messenger RNA (mRNA) purified from total RNA using poly-T oligo-attached magnetic beads. After fragmentation, the first-strand cDNA was synthesized using random hexamer primers, followed by the second-strand cDNA synthesis using dUTP. The library was checked with Qubit and real-time PCR for quantification and a bioanalyzer for size distribution detection. Quantified libraries were pooled and sequenced on Illumina platforms, followed by the clustering of the index-coded samples according to the manufacturer’s instructions. After cluster generation, the library preparations were sequenced on an Illumina platform and paired-end reads were generated. An index of the reference genome (i.e., hg38) was built and reads were aligned to the reference genome using Hisat2 v2.0.5. Differential expression analysis of two conditions/groups (two biological replicates per condition) was performed using the DESeq2 R package (1.20.0). Differential gene expression analysis can be found in Supplementary Dataset S1 and in ArrayExpress (accession number: E-MTAB-14965).

ScRNA-seq using GBM1A and GBM1A-Oct4/Sox2+ cells was performed using the 10X Genomics Chromium v2 platform according to the standard protocol. Reads were sequenced using the Illumina NovaSeq system and aligned using the hg38 genome. Count matrices were generated using CellRanger. Data processing was conducted using the Seurat v5 package in R Studio [19]. Low-quality cells, defined as number of features and/or counts <500 and percentage of mitochondrial reads > 20%, were excluded and the counts were normalized using the SCTransform() function prior to downstream analyses. Batch correction was performed using Harmony [20]. Dimensionality reduction was performed using the first 20 principal components when applicable.

Expression scores for gene signatures in bulk RNA-seq samples were calculated using single-sample gene-set enrichment analysis (Supplementary Table S4) [21]. In scRNA-seq, gene signatures for 6 GBM cellular states (MES1, MES2, AC, OPC, NPC1, and NPC2) were obtained from Neftel et al. [22] and scores were calculated using the AddModuleScore() function in Seurat with the following parameters: nbins = 30 and ctrl = 100. The MES and NPC module scores were calculated by averaging the MES1/MES2 and NPC1/NPC2 values, respectively. To generate the cell state plot, the *y*-axis coordinate for each cell was calculated as y = max(SCopc, SCnpc) − max(SCac, SCmes) where SC = the state module score for a given cell. A positive *y*-axis value indicated OPC/NPC lineage while a negative value indicated AC/MES lineage. The *x*-axis coordinate was then calculated using the following formulas, depending on lineage. For OPC/NPC lineage, x = log2(ABS(SCopc − SCnpc) + 1). For AC/MES lineage, x = log2(ABS(SCac − SCmes) + 1). The custom R scripts used to generate data figures are available upon request.

### 2.8. Analysis of Publicly Available GBM Expression Data

TCGA (HG-U133A) and Rembrandt clinical and transcriptional data from patient glioma and non-tumor brain specimens were obtained from the GlioVis data portal (http://gliovis.bioinfo.cnio.es/ last accessed on 8 April 2025). RNA-seq data from patient-derived xenograft (PDX) cell lines and patient-derived GSC lines were obtained from cBioPortal and GSE119776, respectively. Patient GBM and GSC scRNA-seq data were obtained from the Broad Institute Single Cell Portal (www.singlecell.broadinstitute.org last accessed on 8 April 2025) under study SCP503. A list of the scRNA-seq datasets used for the pan-cancer analysis can be found in Supplementary Table S5. Count matrices for scRNA-Seq analysis were processed as described above and cell annotations, normalized gene expression, and UMAP coordinates from original publications were used for all other downstream analyses. Processed proteomic profiling data from 134 clinical GBM specimens were obtained from Tatari et al. [23] (MassiVE ID: MSV000087947).

### 2.9. Isolation and Activation of PBMC-Derived T Cells

CD4+ and CD8+ T cells were isolated from patient-derived PBMCs [24] using MOJO anti-CD4 and anti-CD8 bead isolation kits (BioLegend, San Diego, CA, USA), respectively. T cells were then activated via anti-CD28/CD3 Dynabeads^TM^ (Thermo Fisher) and recombinant IL2 (Thermo Fisher; 25U per 1 × 10^5^ cells). Activated cells were cultured in RPMI-1640 media containing 2 mM L-glutamine, 10 mM HEPES, 1 mM sodium pyruvate, 4500 mg/L glucose, and 1500 mg/L sodium bicarbonate and supplemented with 10% fetal bovine serum, as recommended by the ATCC.

The effect of GSCs on CD4+ and CD8+ T cells was assessed by culturing the T cells in GSC-conditioned media (CM) for 48–72 h. To generate the CM, GSCs were cultured in neurosphere medium ± ITD-1 (20 μM) or in GSC medium ± doxycycline (1 μg/mL) to induce shTGFBR2 for 48 h and 5 days, respectively. The GSCs were then rinsed and replated at equal densities (~5 × 10^5^ cells/mL) in fresh neurosphere medium (lacking ITD-1 or doxycycline) for 48 h. The CM was then collected and added to immune cells plated at 100–200 K cells/well in 24-well cell culture plates at a 1:1 ratio (*v*/*v*) to immune cell media. The cells were collected to quantify cell viability and measure gene expression changes.

### 2.10. Immune Cell Co-Culture Assay

T cell-mediated tumor killing was assessed using T cells collected after 48 h of culturing in GSC-conditioned medium. CM was collected as described in Section 2.9. Activated CD4^+^ or CD8^+^ T cells (1.0 × 10^5^ cells) were resuspended in a 1:1 ratio (*v*/*v*) of CM/T cell media and added onto GSCs previously seeded onto laminin-coated plates at equal densities (1.0 × 10^5^ cells/well). T cells were removed after 48 h by washing wells with phosphate-buffered saline (PBS) and GSC death was analyzed as described in Section 2.5 above.

### 2.11. Immunofluorescence Imaging

To quantify TGFBR2 knockdown and T cell exhaustion, cells were collected, counted, and then spun onto microscope slides at a density of 100 K cells per spot using cytospin technology. Cells were fixed for 20 min with a 1% paraformaldehyde solution and then washed with PBS before blocking for 1 h with PBS containing 1% bovine serum albumin (BSA) (Sigma-Aldrich). Fluorescence-conjugated primary antibodies (Supplementary Table S3) were added onto cells (1:200–1:500 in 1% BSA-PBS) and incubated at 4 °C overnight. The next day, cells were washed with PBS and coverslips were mounted with Prolong Gold Antifade plus DAPI mounting media (Cell Signaling, Danvers, MA, USA). The slides were imaged with a Leica DMi8 Thunder Imager Live Cell microscope and the fluorescence signal was quantified using ImageJ v1.51 software with background noise removal (NIH). Protein expression was calculated relative to the DAPI signal in each field of view (40× magnification).

### 2.12. Statistical Analyses

All experiments were performed in triplicate and repeated at least twice in each cell model (N ≥ 6). PRISM GraphPad 10 was used to perform all the statistical analyses presented. Two group comparisons were analyzed for variation and significance using a two-tailed, type 1 *t*-test, and *p*-values lower than 0.05 were considered significant and are symbolized by an asterisk in the graphs. One-way ANOVA and Tukey post hoc tests were used to analyze the relationships when comparing multiple experimental groups with *p*-values lower than 0.05 considered statistically significant. All data shown are representative of the mean ± SD of triplicate results unless otherwise specified.

## 3. Results

### 3.1. Oct4 and Sox2 Induce a TGFBR2-Related Mesenchymal Shift in GBM Cells

**Figure 1:**
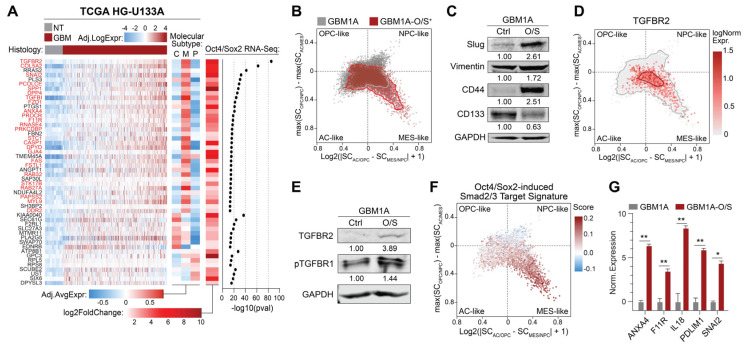
Oct4 and Sox2 drive mesenchymal-like shift in GSCs associated with activation of TGFBR2 signaling. (**A**) Heatmap (left) showing expression of genes upregulated in IDH-wt GBM versus non-tumor (NT) brain tissue, enriched in mesenchymal GBMs (middle), and induced by transgenic co-expression of Oct4/Sox2 in patient-derived GBM neurospheres (right). Genes in red are significantly enriched in mesenchymal GBMs compared to classical and proneural genes. (**B**) Cell state plot of GBM1A neurospheres ± transgenic co-expression of Oct4/Sox2 (O/S). (**C**) Western blot analysis showing expression of mesenchymal driver Slug, mesenchymal markers Vimentin and CD44, and proneural marker CD133. Numerical values represent band signal intensity relative to parental (Ctrl) cells and normalized to GAPDH. (**D**) Cell state plot showing TGFBR2 expression in GBM1A and GBM1A-O/S neurospheres. (**E**) Western blot showing expression of TGFBR2 and phospho-TGFBR1 in GBM1A cells ± co-expression of Oct4/Sox2. Numerical values represent band signal intensity relative to parental (Ctrl) cells and normalized to GAPDH. (**F**) Cell state plot showing expression Smad2 and/or Smad3 transcriptional targets (GSE11710) induced by Oct4/Sox2 in GBM1A cells ± O/S. (**G**) qRT-PCR analysis comparing expression of subset of Smad2/Smad3 targets in GBM1A cells ± O/S. Parental GBM1A cells were used as controls. Data are shown as mean ± SD. Statistical significance was calculated using Student’s *t*-test; see panel (**G**). * *p* < 0.05; ** *p* < 0.01. Data shown in panels (**C**,**D**) are representative of 2 independent experiments; experiments shown in panel G were performed in triplicate and are representative of 2 independent experiments.

To explore clinically relevant Oct4/Sox2-induced reprogramming events in GBM, we began by conducting bulk RNA sequencing (RNA-seq) on patient-derived neurospheres with and without the transgenic co-expression of Oct4 and Sox2, reprogramming transcription factors shown by us and others to induce tumor-propagating GSC phenotypes in GBM cells [5,9]. We then cross-referenced the resulting differential gene expression data to transcripts upregulated in clinical GBM specimens compared to non-tumor tissue. This analysis identified a collection of genes both induced by Oct4 and Sox2 and enriched in GBM compared to non-tumor brain tissue (Figure 1A). Furthermore, 25 of these genes were found to be significantly upregulated in mesenchymal GBMs versus other molecular subtypes, and of these genes, *TGFBR2* ranked the highest (Figure 1A and Supplementary Table S6; genes in red).

Single-cell RNA sequencing (scRNA-seq) analysis conducted on patient-derived GBM neurospheres enriched with GSCs ± transgenic Oct4/Sox2 showed a shift towards the MES-like cell state and away from the OPC-like, NPC-like, and AC-like neurodevelopmental phenotypes (Figure 1B) [22]. Consistent with these transcriptomic signatures, Western blot analysis showed that Oct4/Sox2 expression increased the mesenchymal protein markers Slug, Vimentin, and CD44 concurrently with the decreased expression of the proneural marker CD133 [6,25] (Figure 1C). *TGFBR2* was also most strongly associated with MES-like GBM cells relative to other neurodevelopmental subtypes (Figure 1D), consistent with its upregulation by Oct4/Sox2 and association with clinical mesenchymal GBM (Figure 1A). Western blot analysis confirmed the upregulation of both TGFBR2 and phosphorylated TGFBR1, a surrogate for activated TGFβ signaling, in neurospheres expressing transgenic Oct4 and Sox2 (Figure 1E). Moreover, a set of ChIP-validated [26] Smad2/3 transcriptional targets were preferentially induced by Oct4/Sox2 in mesenchymal-like neurospheres (Figure 1F), and qRT-PCR analysis confirmed the upregulation of a subset of Smad2/3 target genes by Oct4/Sox2 co-expression (Figure 1G).

**Figure 2:**
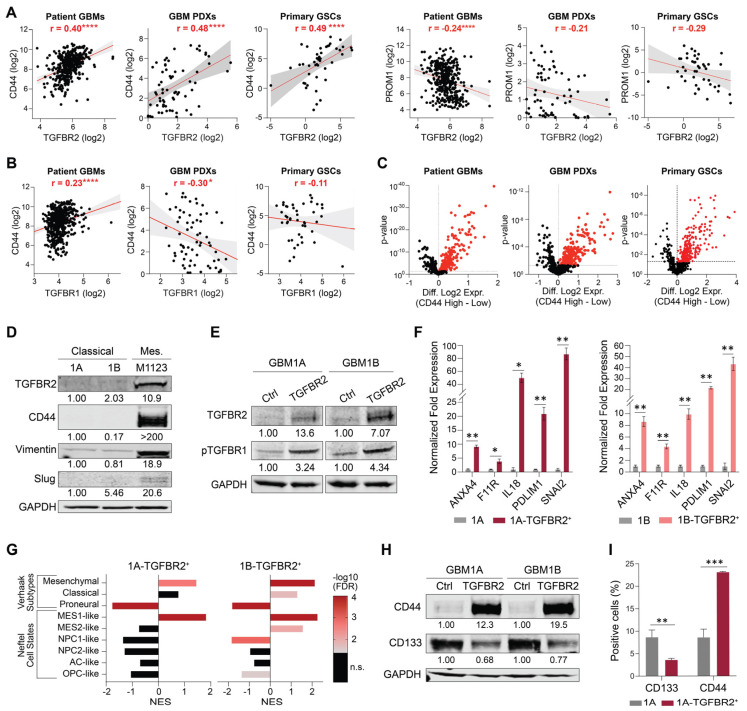
TGFBR2 is sufficient to induce mesenchymal shift in GSCs. (**A**) Pearson correlation plots comparing mRNA expression of CD44 (left) and PROM1 (right) to TGFBR2 in GBM patient specimens (TCGA HG-U133A), GBM patient-derived xenograft (PDX) lines, and primary patient-derived GSCs. (**B**) Pearson correlation plots comparing mRNA expression of CD44 to TGFBR1 in GBM patient specimens, GBM PDXs, and primary GSCs. (**C**) Volcano plots showing expression of Smad2/3 transcriptional targets in high-CD44 vs. low-CD44 samples from GBM patient specimens, GBM PDXs, and primary GSCs. (**D**) Western blot comparing expression of TGFBR2 and mesenchymal markers CD44, Vimentin, and Slug in GBM1A (1A), GBM1B (1B), and M1123 patient-derived neurospheres. Numerical values represent band signal intensity relative to GBM1A and normalized to GAPDH. (**E**) Western blot showing expression levels of FLAG (TGFBR2) and phospho-TGFBR1 in GBM neurospheres ± transgenic FLAG-tagged TGFBR2. Numerical values represent band signal intensity relative to parental line (GBM1A or GBM1B) and normalized to GAPDH. (**F**) qRT-PCR analysis showing expression of subset of Smad2/3 transcriptional targets in GBM neurospheres ± transgenic TGFBR2. Parental GBM1A or GBM1B cells were used as controls. (**G**) GSEA of GBM molecular subtypes from TGFBR2-induced transcriptomes. Color of bars represent false discovery rate (n.s. = not significant). (**H**) Western blot showing CD44 and CD133 protein levels in neurospheres ± transgenic TGFBR2. Numerical values represent band signal intensity relative to parental (Ctrl) cells and normalized to GAPDH. (**I**) Flow cytometry analysis to measure CD44+ and CD133+ cell populations following transgenic expression of TGFBR2 in GSCs. Data in panels (**F**,**I**) are shown as mean ± SD. Statistical significance was calculated using Pearson’s correlation in panels (**A**,**B**) and Student’s *t*-test in panels (**C**,**F**,**I**). * *p* < 0.05; ** *p* < 0.01; *** *p* < 0.001, **** *p* < 0.0001. Data shown in panels (**D**,**E**,**H**) are representative of 2 independent experiments; experiments shown in panel (**F**) were performed in triplicate and are representative of 2 independent experiments.

Further analyses of clinical, patient-derived xenograft lines and primary GSC transcriptomic data revealed a consistent and strong positive correlation between TGFBR2 expression and the mesenchymal marker *CD44* across these diverse datasets, contrasting the weak and negative correlations with the proneural marker CD133 (*PROM1*) (Figure 2A). Notably, *TGFBR1* expression did not positively correlate with *CD44* across the three complementary clinical, PDX, and primary GSC datasets (Figure 2B). Separating these same datasets into high/low based on median *CD44* expression showed that Smad2/3 transcriptional targets were enriched in high-*CD44* samples (Figure 2C). These correlations were confirmed by Western blot analysis showing higher endogenous levels of TGFBR2 in mesenchymal patient-derived neurospheres compared to more classical neurospheres (Figure 2D). Together, these observations predict that TGFBR2 signaling plays a prominent role in mesenchymal transitions driven by reprogramming events initiated by Oct4 and Sox2 in GBM. To test the hypothesis that TGFBR2 signaling is sufficient to drive a mesenchymal transition, we expressed transgenic TGFBR2 in classical GBM1A and GBM1B neurospheres that endogenously expressed low levels of TGFBR2 (Figure 2D). The transgenic expression of TGFBR2 was sufficient to activate TGFβ signaling, as measured by TGFBR1 phosphorylation (Figure 2E) and the expression of downstream Smad2/3 transcriptional targets (Figure 1G and Figure 2F).

Recent advances in our molecular understanding of GBM show that cells within the tumor exist in four main cellular states: three that recapitulate neural cell types (i.e., astrocyte-like, oligodendrocyte-like, and neural progenitor-like types) and a fourth that resembles mesenchymal cells (i.e., mesenchymal-like type) [22]. These GBM molecular subtypes correlate with disease outcomes, with the mesenchymal state linked to a more invasive and aggressive GBM phenotype, higher immune infiltration, and poorer prognosis [27,28]. Unbiased transcriptome analyses via RNA-Seq showed that TGFBR2 expression enriched mesenchymal signatures and depleted proneural, NPC-like, AC-like, and OPC-like signatures in these cell populations (Figure 2G). Consistently with mesenchymal induction, transgenic TGFBR2 induced a shift towards a high-CD44, low-CD133 GSC state as determined by the Western blot and flow cytometry (Figure 2H,I). Collectively, these results demonstrate that TGFBR2 is sufficient to induce a mesenchymal shift in GSCs.

### 3.2. TGFBR2 Induces an Immunosuppressive ITL Signature in Mesenchymal GSCs

**Figure 3:**
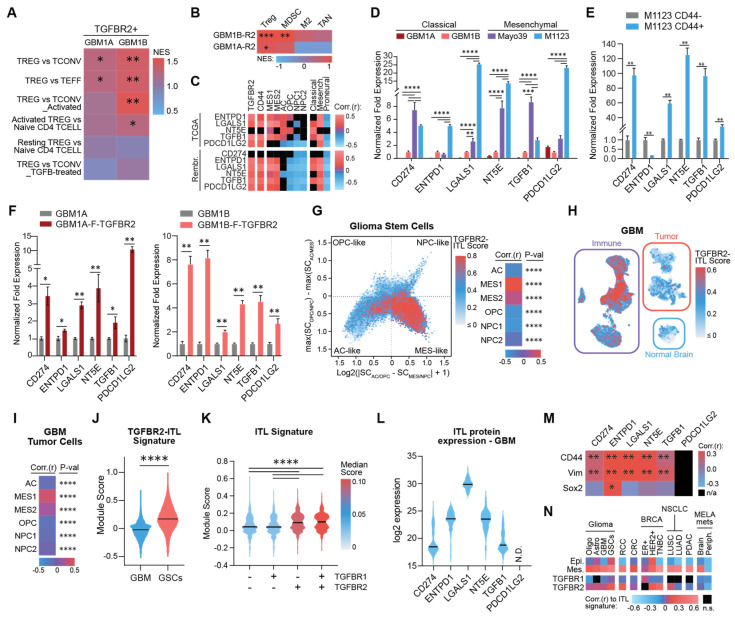
TGFBR2 induces immunosuppressive Treg-like signature in mesenchymal GSCs. (**A**) Heatmap of GSEA showing enrichment of Treg-related genes in GSCs expressing transgenic TGFBR2. NES = normalized enrichment score. Parental GBM1A or GBM1B cells were used as controls. (**B**) Heatmap of GSEA showing enrichment scores for gene signatures for immunosuppressive cell types in GSCs expressing transgenic TGFBR2. MDSC = myeloid-derived suppressor cell; M2 Macro = M2-polarized macrophage; TAN = tumor-associated neutrophil. (**C**) Heatmap showing Pearson’s coefficient values comparing Treg effector genes and TGFBR2 and CD44 GBM cell states and GBM molecular subtypes in clinical GBM specimens. TCGA = TCGA HG-U133A; Rembr. = Rembrandt. (**D**) qRT-PCR analysis measuring expression of Treg effector genes in GBM neurospheres. (**E**) qRT-PCR analysis comparing expression of Treg effector genes in CD44+ versus CD44- GSCs. (**F**) qRT-PCR analysis comparing expression of Treg effector genes in GSCs ± transgenic TGFBR2. Parental GBM1A or GBM1B cells were used as controls (**G**) Cell state plot (left) showing expression of TGFBR2-ITL signature in GBM cells enriched with GSCs derived from 26 patient tumors [37]. Heatmap showing Pearson’s coefficient (right) from same cells showing correlations with GBM cell states. (**H**) UMAP showing expression of TGFBR2-ITL signature in scRNA-seq from clinical GBM specimens. Single-cell data from 7 patient GBMs were obtained from Richards et al. [37]. (**I**) Heatmap showing Pearson’s coefficient from tumor cells in panel H showing correlations with GBM cell states. (**J**) Frequency distribution plot of TGFBR2-ITL gene signature score in tumor cells from clinical GBM specimens compared to patient-derived GSCs. (**K**) Frequency distribution plot of ITL gene signature score in patient-derived GSCs grouped by ± expression of TGFBR1 and TGFBR2. (**L**) Violin plot showing protein expression of ITL genes in GBM clinical specimens. Protein expression was retrieved from Mass Spectrometry Interactive Virtual Environment (MassIVE) with following MassiVE ID: MSV000087947. (**M**) Heatmap showing Pearson coefficients between ITL genes protein expression and proneural marker Sox2 or mesenchymal markes CD44 and Vimentin (Vim) in GBM clinical specimens. (**N**) Heatmap showing Pearson’s coefficient from pan-cancer analysis showing correlations between ITL signature and TGFBR1, TGFBR2, and epithelial (Epi.) and mesenchymal (Mes.) signatures in various cancer types. Oligo = oligodendroglioma; Astro = astrocytoma; RCC = renal cell carcinoma; CRC = colorectal cancer; BRCA = breast cancer; TNBC = triple-negative breast cancer; NSCLC = non-small cell lung cancer; LUSC = lung squamous cell carcinoma; LUAD = lung adenocarcinoma; PDAC = pancreatic ductal adenocarcinoma; MELA mets = melanoma metastases; Periph. = peripheral; n.s. = non-significant. Data in panels (**D**–**F**) are shown as mean ± SD. Statistical significance was calculated using nominal *p*-value in panels (**A**,**B**), Pearson’s correlation in panels (**C**,**G**,**I**,**L**), one-way ANOVA with Tukey’s post hoc test in panel (**D**), Student’s *t*-test in panels (**E**,**F**), Mann–Whitney U-test in panel J, and Kruskal–Wallis test in panel (**K**). * *p* < 0.05; ** *p* < 0.01; *** *p* < 0.001; **** *p* < 0.0001. Experiments shown in panels (**E**,**F**) were performed in triplicate and are representative of 2 independent experiments.

To identify novel molecular events driven by TGFBR2 signaling in GSCs, we performed an unbiased gene-set enrichment analysis (GSEA) on the genes differentially induced by transgenic TGFBR2. Interestingly, this analysis revealed the enrichment of several gene signatures related to the Treg phenotype (Figure 3A). To determine if these gene signature changes were specific to the Treg phenotype, we performed GSEA using gene signatures corresponding to multiple immunosuppressive cell types typically found in the GBM immune TME (i.e., Tregs, myeloid-derived suppressor cells (MDSCs), suppressive M2-like macrophages, and tumor-associated neutrophils) [29,30,31,32,33,34,35]. The TGFBR2-induced transcriptome was most consistently and significantly associated with the Treg signature, inconsistently associated with the MDSC signature, and unassociated with either the macrophage or neutrophil signatures (Figure 3B). We identified 362 unique genes upregulated by transgenic TGFBR2 in GBM neurospheres and associated with a Treg state (Supplementary Table S7), consistent with the hypothesis that TGFBR2 mediates an immunosuppressive mesenchymal shift that resembles Treg functionality in GSCs. Tregs reprogram the immune TME by inhibiting anti-tumor immune cell function in a variety of ways including releasing cytokines and proteins and signaling through ligand–receptor interactions and ectoenzymes on the cell surface [36]. To identify genes that may play a direct immunosuppressive role in mGSCs, we first queried a scRNA-seq dataset of patient-derived GSCs for genes known to be direct immunosuppressive effectors in Tregs. Using this approach, we identified *CD274* (PD-L1), *NT5E* (CD73), *ENTPD1* (CD39), *LGALS1* (galectin-1), *PDCD1LG2* (PD-L2), and *TGFB1* as putative immunosuppressive factors in this cell subset (Figure S1). Of note is that we did not detect transcripts for *FOXP3*, *CD25*, and *IL2R*, master regulators of Treg development and function, or *IL10* and *Granzyme B* [36]. Analysis of clinical GBM transcriptomic datasets (TCGA and Rembrandt) showed a positive correlation between the six immunosuppressive genes identified and the TGFBR2^High^, CD44^High^, and mesenchymal GBM cell subsets, as defined by Neftel et al. and Verhaak et al. [22,27] (Figure 3C). We refer to this gene set as the immunosuppressive Treg-like (ITL) signature.

Consistently with these transcriptomic associations, we measured higher expressions of the ITL-signature genes in mGSCs compared to classical neurosphere isolates (Figure 3D and Figure S2) and five out of six genes were enriched in CD44+ cells compared to their CD44- counterparts (Figure 3E). Additionally, we determined that the transgenic expression of TGFBR2 was sufficient to induce the mRNA expression of all six genes in the ITL signature in two distinct classical neurosphere isolates (Figure 3F). As predicted, this TGFBR2-induced ITL (TGFBR2-ITL) gene signature was highly expressed in MES-like patient-derived GBM cells enriched with GSCs (Figure 3G). Critically, this signature was also found to be expressed in neoplastic tumor cells within clinical GBM-pathology specimens (Figure 3H) and was specifically enriched in the MES-like GBM cell subset (Figure 3I). This TGFBR2-driven ITL gene signature had a significantly higher expression in patient GSCs compared to all neoplastic GBM cells within patient tumors (Figure 3J), emphasizing the enrichment of this signature in stem-like cells. Notably, this ITL signature did not correlate with TGFBR1 expression, indicating the distinct role of TGFBR2 in controlling the ITL GSC phenotype (Figure 3K). We also found that GBM clinical specimens expressed detectable levels of protein for *CD274* (PD-L1), *NT5E* (CD73), *ENTPD1* (CD39), *LGALS1* (galectin-1), and *TGFB1* but not *PDCD1LG2* (PD-L2) (Figure 3L). Similarly to what was determined by our transcriptomic analyses, we also measured a positive correlation between the mesenchymal markers CD44 and Vimentin and the ITL genes but not the proneural marker Sox2 in the same proteomic dataset (Figure 3M). Interestingly, TGFBR1, TGFBR2, CD133, and Ascl1 expressions were not captured in this proteomic dataset. Furthermore, pan-cancer bioinformatics analysis revealed a strong association between the six-gene ITL signature and both mesenchymal signatures and TGFBR2 expression in a variety of systemic cancers (Figure 3N). Together, these results identified the TGFBR2-induced signature of immunosuppressive effector genes, known to be expressed by Tregs, in mesenchymal cancer cells across multiple solid tumor types.

### 3.3. TGFBR2 Inhibition Blocks the Immunosuppressive GSC Phenotype

**Figure 4:**
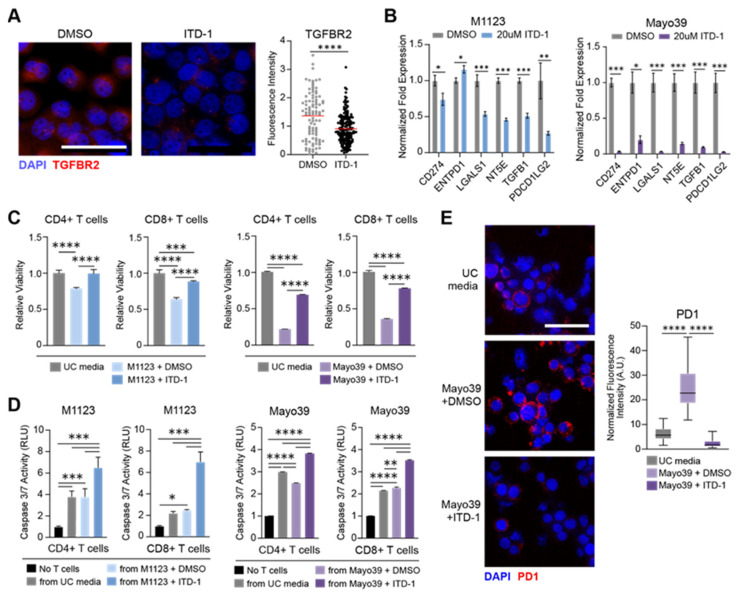
Pharmacological TGFBR2 inhibition attenuates immunosuppressive phenotype of GSCs. (**A**) Representative immunofluorescence images (left) and quantification (right) showing TGFBR2 expression in M1123 cells 24 h after treatment with ITD-1 (20 μM) or vehicle control (DMSO). Scale bar = 50 μm. (**B**) qRT-PCR analysis showing expression of Treg effector genes 72 h following treatment with ITD-1 (20 μM) or vehicle control (DMSO). (**C**) CD4+ and CD8+ T cell viability was measured 48 h after culture in media conditioned with GSCs treated with ITD-1 or DMSO. CD4+ and CD8+ T cells cultured in unconditioned (UC) media were used as control. (**D**) Tumor cell death was measured via caspase 3/7 assay 48 h after co-culturing with CD4+ or CD8+ T cells cultured in media conditioned with GSCs pre-treated with ITD-1 or DMSO as described in Materials and Methods. Tumor cells cultured in absence of CD4+ or CD8+ T cells were used as baseline control. (**E**) Representative immunofluorescence images (left) and quantification (right) of PD1 expression in CD8+ T cells 48 h after culturing in media conditioned with GSCs treated with ITD-1 or DMSO. Scale bar = 50 μm. PD1 fluorescence intensity was measured using ImageJ v1.51 ‘Analyze Particles’ feature and normalized to total DAPI intensity in multiple fields of view (n = 20 per condition). Statistical significance was calculated using Mann–Whitney U-test for panel A, Student’s *t*-test for panel (**B**), one-way ANOVA with Tukey’s post hoc test for panels (**C**–**E**). Data are shown as mean ± SD for all bar graphs. * *p* < 0.05; ** *p* < 0.01; *** *p* < 0.001; **** *p* < 0.0001. Data shown in panels (**B**–**E**) were gathered in triplicate and are representative of 2 independent experiments.

Our results showed that TGFBR2 induced a subset of immunosuppressive effectors associated with Treg function, predicting that TGFBR2 inhibition would reduce the immunosuppressive capacity of GSCs. To test this, we utilized Inducer of TGFBR2 Degradation-1 (ITD-1), a small-molecule TGFBR2 inhibitor that activates proteasome-dependent TGFBR2 protein degradation [38] and GBM cells engineered for doxycycline-induced shRNA-mediated TGFBR2 expression knockdown. Quantitative immunofluorescence analysis confirmed that ITD-1 depleted TGFBR2 protein (Figure 4A) and qRT-PCR showed that shTGFBR2 inhibited TGFBR2 expression in patient-derived mGSCs (Figure S3A). ITD-1 and shTGFBR2 also inhibited the expression of the six-gene ITL signature in patient-derived mGSCs (Figure 4B and Figure S3B). These results showed that TGFBR2 signaling was required to maintain the expression of the six-gene ITL signature and predicted that ITD-1 and shTGFR2 would reduce the immunosuppressive phenotype of mGSCs. To test these predictions, we examined the effects of conditioned medium (CM) obtained from mGSCs ± TGFBR2 inhibition on the viability and tumor cell-killing capacity of PBMC-derived CD4+ and CD8+ T cells. Compared to the effect of unconditioned medium, medium conditioned by DMSO-treated mGSCs significantly reduced the viability of CD4+ and CD8+ T cells. The capacity of CM to inhibit T cell viability was lost if collected from mGSCs pre-treated with ITD-1 or from TGFBR2-knockdown GBM cells (Figure 4C and Figure S3C). CD4+ and CD8+ T cells cultured in CM obtained from mGSCs pre-treated with ITD-1 or from TGFBR2-knockdown GBM cells displayed enhanced TCR-independent tumor cell-killing capacity compared to T cells cultured in either unconditioned medium or in CM from control mGSCs (DMSO-treated or no doxycycline) (Figure 4D and Figure S3F). We further investigated the impact of inhibiting GBM-cell TGFBR2 on the CD8+ T cell expression of the exhaustion marker PD-1. CM from mGSCs markedly induced the CD8+ T cell expression of PD-1, an effect completely abrogated by inhibiting the neurosphere cell TGFBR2 with either ITD-1 or shTGFBR2 prior to CM collection (Figure 4E and Figure S3D). Collectively, these results demonstrate that the selective targeting of TGFBR2 represses the ITL gene signature in GSCs and inhibits their immunosuppressive effects on CD4+ and CD8+ T cells.

## 4. Discussion

The immunosuppressive TME is a hallmark of GBM, and GBMs characterized by a mesenchymal transcriptome contain an especially high proportion of immunosuppressive cells along with having the shortest median survival and reduced sensitivity to standard-of-care therapy relative to other molecular subtypes [28,39]. Tumor cells are known modulators of the immune GBM TME, with GSCs having potent immunosuppressive capacity. Recent studies have highlighted multiple GSC-specific factors responsible for recruiting and polarizing TAMs to a pro-tumor M2-like phenotype [40,41,42] or suppressing anti-tumor T cell infiltration and function [3,43]. However, the extent to which GSC-intrinsic mechanisms impede anti-tumor immune cell function and the targetable factors responsible are not fully understood. Our lab recently described how stem cell-driving events coordinated by Oct4 and Sox2 enhance tumorigenicity and tumor cell-mediated immunosuppression [5,9]. In particular, Oct4 and Sox2 upregulate the expression of certain immune checkpoint molecules, cytokines, and chemokines in a BRD4-dependent manner, resulting in enhanced T cell apoptosis, Treg infiltration, and immunosuppressive M2-like macrophage polarization [5]. We now show that stem cell-reprogramming events initiated by Oct4 and Sox2 induce a mesenchymal transition in GBM cells characterized by the activation of TGFBR2 signaling (Figure 1 and Figure 2) which, in turn, mediates transcriptome changes resembling a Treg state (Figure 3 and Figure S1). Critically, the immunosuppressive phenotype of TGFBR2^High^ mGSCs is blocked by TGFBR2 inhibition, decreasing CD8+ T cell exhaustion and restoring CD4+ and CD8+ T cell tumor cell-killing ability in vitro (Figure 3, Figure 4 and Figure S3).

Previous research shows that tumor cells are capable of co-opting developmental pathways associated with non-neoplastic cells to support tumor growth and therapeutic resistance [44]. In GBM, tumor cells have demonstrated the mimicry of vascular cells [45] and neuronal- and glial-progenitor cells [46,47]. Moreover, GSCs can acquire a myeloid-like transcriptional profile following repeated immune exposure, facilitating immune evasion and promoting the infiltration and polarization of pro-tumor myeloid cells [48]. We now show that TGFBR2 is associated with and activates a Treg-like state as GSCs become more mesenchymal and immunosuppressive (Figure 2, Figure 3, Figure 4 and Figure S1). This recapitulates, to some extent, the capacity of TGFβ signaling to differentiate naïve T cells into an induced Treg state [49] and further delineates its role in the acquisition of immunosuppressive cell states. Similarly to previous reports describing Foxp3- CD103+ T cell-derived induced Tregs [50], the absent expression of the canonical Treg markers Foxp3 and CD25 in the GSC-derived induced Treg-like cells described here suggests that GSCs do not transdifferentiate into Tregs in response to TGFBR2 but instead co-opt certain mechanisms utilized by Tregs to exert immunosuppressive behavior. Among the multitude of genes induced by TGFBR2, we identified a six-gene signature comprising putative direct effectors of the immunosuppressive GSC phenotype (Figure 3). These include *NT5E* (CD73) and *ENTPD1* (CD39) which are involved in immunosuppressive adenosine signaling [51], *LGALS1* (galectin-1) and *TGFB1*, which encode for anti-inflammatory cytokines [52,53], and *CD274* (PD-L1), which binds to PD-1 to initiate an inhibitory signaling pathway in anti-tumor immune cells [54]. Notably, this molecular phenotype correlates with previously defined cell states in GBM associated with immune cell interaction and an immunosuppressive TME (Figure 3) [22,55]. We also show that the correlation of this immunosuppressive signature with *TGFBR2* and the mesenchymal state is conserved in a variety of systemic cancers (Figure 3N), suggesting that the findings from this study may be applicable to multiple tumor types.

Although blocking TGFβ signaling is widely viewed as a promising anti-tumor strategy, efforts in GBM have mainly focused on blocking TGFBR1 activity without successful clinical translation [56,57,58]. This might be explained by our current results specifically identifying TGFBR2 as the driver of GSC-derived induced Treg-like cells and associating their immunosuppressive signature with *TGFBR2* but not *TGFBR1* expression in clinical specimens across multiple cancers, uncovering previously unrecognized TGFBR2 dependencies in cancer (Figure 3K,N). Currently, the only TGFBR2-specific drug that has been tested in a clinical setting is IMC-TR1 (LY3022859), an anti-TGFBR2 monoclonal antibody, which showed no efficacy in a Phase 1 trial for advanced solid tumors [59]. Despite the limited efficacy of these inhibitors, alternative approaches employing combinations with immunotherapies still hold promise [60]. We showed that blocking TGFBR2 via an shRNA or a selective inhibitor, ITD1 [38], reduced the expression of this gene signature in mGSCs (Figure 4C and Figure S3B) and we demonstrated through T cell culture assays that mGSCs expressing this ITL transcriptional signature reduced CD4^+^ and CD8^+^ T cell viability and function, which could be rescued by blocking TGFBR2 signaling (Figure 4 and Figure S3).

## 5. Conclusions

**Figure 5:**
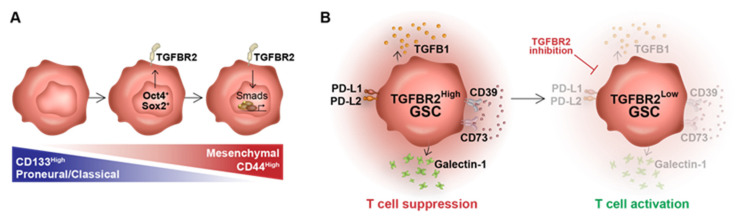
Graphic summary. (**A**) Glioma stem cells in a proneural, CD133^high^ state can transition to a mesenchymal, CD44^high^ state following the co-expression of the reprogramming transcription factors Oct4 and Sox2. Oct4 and Sox2 induce the expression of TGFBR2, activating downstream signaling and promoting a mesenchymal state. (**B**) High TGFBR2 expression leads to the upregulated expression of immunosuppressive effector genes to generate a T cell-suppressing GSC phenotype. TGFBR2 inhibition attenuates the expression of these effectors, allowing for T cell activation.

In summary, we describe a mechanism by which stem cell-driving events coordinate the transition to a mesenchymal-like GSC state through the activation of TGFBR2 signaling (Figure 5A). In turn, TGFBR2 induces an immunosuppressive GSC phenotype reminiscent of Tregs that allows GSCs to inhibit T cell function by decreasing proliferation capacity and inducing exhaustion (Figure 5B). Blocking TGFBR2 signaling in mGSCs, through both molecular and pharmacological methods, counters this GSC-mediated immunosuppression, predicting that TGFBR2 blockades could cooperate with current immunotherapy to enhance anti-tumor effects in GBM.

## Data Availability

The data supporting this study are available within the paper and its Supplementary Data file. All other data are available from the authors upon reasonable request.

## Supplementary Materials

The following supporting information can be downloaded at https://www.mdpi.com/article/10.3390/cells14080592/s1. Figure S1: Canonical regulatory T cell effector genes are expressed in TGFBR2+ patient-derived GSCs; Figure S2: TGFBR2 induces expression of PDL1 and TGFB1 in GSCs; Figure S3: ShRNA-mediated TGFBR2 knockdown in GSCs attenuates the immunosuppressive ITL phenotype; Supplementary Table S1: Lentiviral constructs used in this study for stable cell line generation; Supplementary Table S2: List of antibodies used in this study; Supplementary Table S3: Primer sequences used in this study for qRT-PCR analyses; Supplementary Table S4: Source information for gene sets used in this study; Supplementary Table S5: Source information for publicly available single-cell RNA-sequencing datasets used in this study; Supplementary Table S6: Genes induced by Oct4 and Sox2 upregulated in GBM; Supplementary Table S7: Treg-related genes induced by transgenic TGFBR2 in GSCs.
