# Supplementary material for: Regulatory T Cell Mimicry by a Subset of Mesenchymal GBM Stem Cells Suppresses CD4 and CD8 Cells
Source: Cells. 2025 Apr 14;14(8):592. doi: 10.3390/cells14080592 (PMC12026101; doi:10.3390/cells14080592)
Supplement: Supplementary file 1 [file cells-14-00592-s001.zip › TGFBR2_ALJ_Supplemental_Cancers_Revised.pdf]

Regulatory T Cell Mimicry by a Subset of Mesenchymal GBM Stem Cells Suppresses CD4 and CD8 Cells

Amanda L. Johnson [1,2], Harmon S. Khela [1], Jack Korleski [1,3], Sophie Sall [1], Yunqing Li [1,2], Weiqiang Zhou [4],
Karen Smith-Connor [1], John Laterra [1,2,5,6,7,*] and Hernando Lopez-Bertoni [1,2,7,*]

[1] Hugo W. Moser Research Institute at Kennedy Krieger, Baltimore, MD 21205, USA; amanda_johnson1@dfci.harvard.edu (A.L.J.); harmon.khela@pennmedicine.upenn.edu (H.S.K.); korleski.john@mayo.edu (J.K.); salls@kennedykrieger.org (S.S.); liyu@kennedykrieger.org (Y.L.); connorka@kennedykrieger.org (K.S.-C.)
[2] Department of Neurology, Johns Hopkins University School of Medicine, Baltimore, MD 21205, USA
[3] Department of Internal Medicine, Mayo Clinic, Rochester, MN 55905, USA
[4] Department of Biostatistics, Johns Hopkins Bloomberg School of Public Health, Baltimore, MD 21205, USA; wzhou14@jhu.edu
[5] Department of Oncology, Johns Hopkins University School of Medicine, Baltimore, MD 21205, USA
[6] Department of Neuroscience, Johns Hopkins University School of Medicine, Baltimore, MD 21205, USA
[7] Sidney Kimmel Comprehensive Cancer Center at Johns Hopkins, Baltimore, MD 21205, USA
* Correspondence: laterra@kennedykrieger.org (J.L.); lopezbertoni@kennedykrieger.org (H.L.-B.)

This PDF file includes:

Figures. S1–S3
Supplementary Tables S1–S7

Supplemental Data

Supplemental Figures

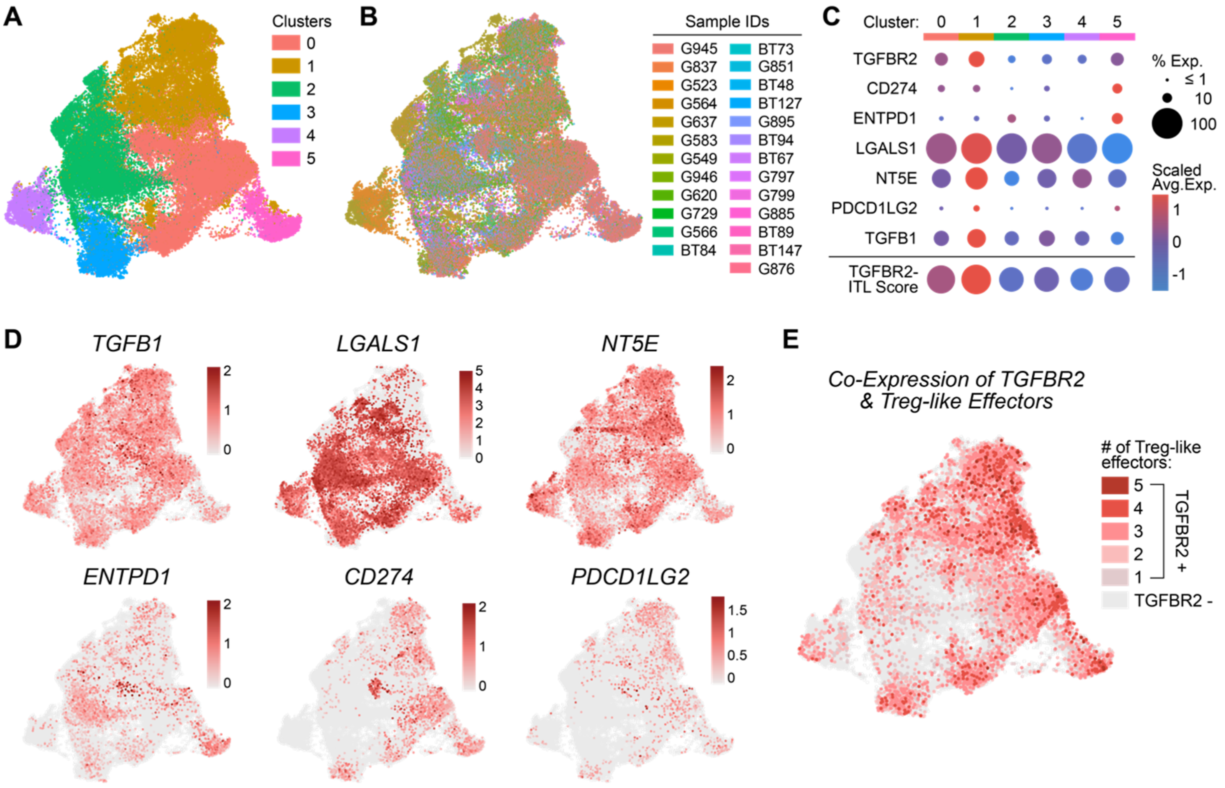

Supplemental Figure S1. Canonical regulatory T cell effector genes are expressed in TGFBR2+ patient-derived GSCs. (A) UMAP showing clustering of patient-derived GSCs. **(B)** UMAP showing distribution of patient samples following Harmony batch correction. **(C)** Dot plot showing expression levels of TGFBR2, regulatory T cell genes, and the TGFBR2-ITL gene signature in cell clusters from patient-derived GSC scRNA-seq. **(D)** UMAPs showing expression of individual Treg-related effector genes in patient-derived GSCs. **(E)** UMAP showing degree of co-expression of Treg-related effectors with TGFBR2 in patient-derived GSCs.

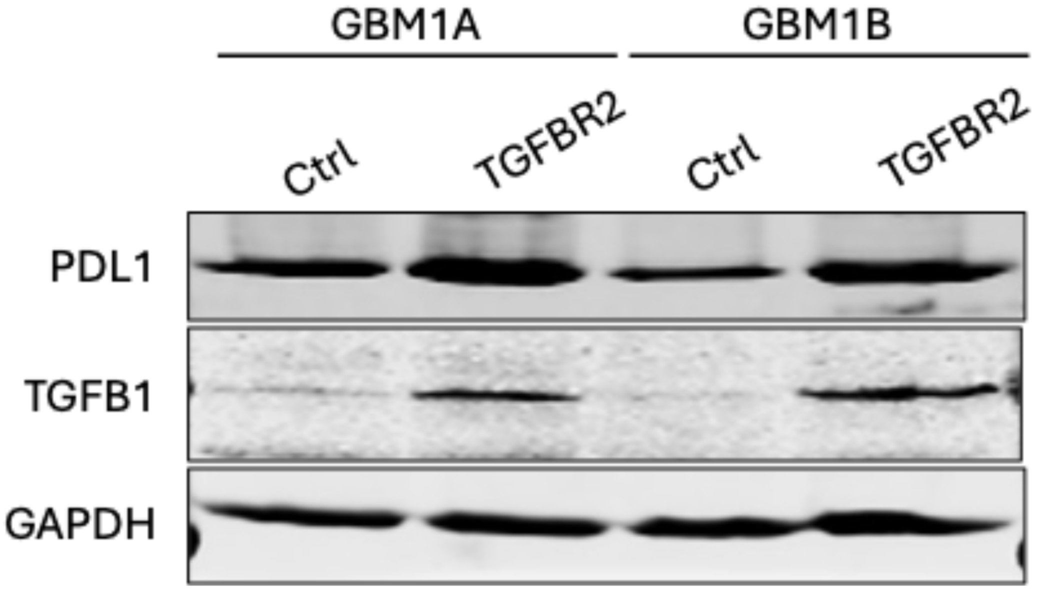

Supplemental Figure S2. TGFBR2 induces expression of PDL1 and TGFB1 in GSCs. Western blot analysis showing protein expression of PDL1 and TGFB1 in GSCs stably expressing transgenic TGFBR2.

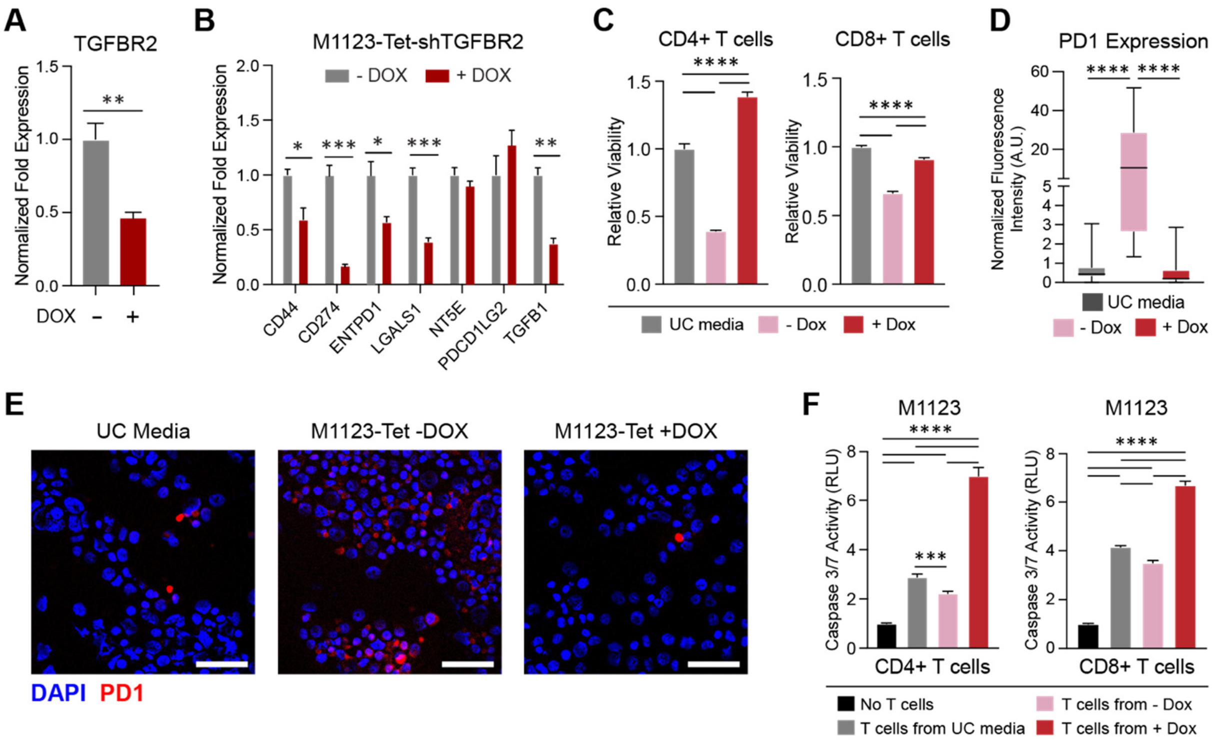

Supplemental Figure S3. ShRNA-mediated TGFBR2 knockdown in GSCs attenuates the immunosuppressive ITL phenotype. (A) qRT-PCR analysis showing knockdown of TGFBR2 expression in M1123-Tet-shTGFBR2 cells 72h following doxycycline (Dox) treatment. **(B)** qRT-PCR analysis showing knockdown of Treg effector genes in M1123-Tet-shTGFBR2 cells 5-days following Dox. **(C)** T cell viability assays showing an increase in cell viability when CD4+ or CD8+ T cells are cultured in media conditioned by M1123 cells with TGFBR2 inhibition (+ Dox) compared to control (-Dox). UC media = unconditioned media. **(D)** Quantification and **(E)** representative immuno-fluorescence images of PD1 expression in CD8+ T cells following culture in media conditioned by M1123-Tet-shTGFBR2 cells -/+ Dox. Quantification was performed from immunofluorescence images and calculated in multiple fields of view (40X magnification, n=20-30 per condition) and normalized to total DAPI signal. Scale bar = 50µm. **(F)** Tumor cell death assays showing enhanced T cell-mediated GSC death following culture in media conditioned by GSCs with TGFBR2 inhibition (+ Dox) compared to controls. Statistical significance was calculated using Student's T-test for panels A & B, one-way ANOVA with Tukey's post hoc test for panels C & F, and Kruskal-Wallis test for panel D. Data are shown as mean ± SD for all bar graphs. *p<0.05, **p<0.01, ***p<0.001, ****p<0.0001.

Supplemental Tables

Supplementary Table S1. Lentiviral constructs used in this study for stable cell line generation.

Lentiviral Vectors		
Construct	*Manufacturer*	*Catalog no.*
pEZX-Flag-TGFBR2	GeneCopoeia	EX-Z4152-Lv242
pTRIPz-shTGFBR2	Dharmacon	V3THS_406963
pLM-vexGFP-Oct4	Addgene	#22240
pLM-mCitrine-Sox2	Addgene	#23242

Supplementary Table S2. List of antibodies used in this study.

**Antibodies**			
Target	*Application (Dilution)*	*Manufacturer*	*Catalog no.*
GAPDH	Western Blot (1:5000)	Santa Cruz Biotechnology	Sc-47724
Slug	Western Blot (1:1000)	Cell Signaling Technology	9585T
Vimentin	Western Blot (1:1000)	Cell Signaling Technology	5741T
CD44	Western Blot (1:1000)	Cell Signaling Technology	156-C311
CD133	Western Blot (1:1000)	GeneTex	GTX102109
TGFBR2	Western Blot (1:500)	GeneTex	GTX129909
Phospho-TGFBR1	Western Blot (1:1000)	Invitrogen	PA5-40298
Flag	Western Blot (1:1000)	Sigma-Aldrich	F1804
CD44	Flow Cytometry (1:100)	Miltenyi Biotec	REA690
CD133	Flow Cytometry (1:100)	Miltenyi Biotec	293C3
TGFBR2	Immunofluorescence (1:200)	R&D Systems	FAB241A
CD279 (PD1)	Immunofluorescence (1:200)	Miltenyi Biotec	REA1165

Supplementary Table S3. Primer sequences used in this study for qRT-PCR analyses.

**qRT-PCR Primers**		
Target Gene	*Forward*	*Reverse*
18S	ACAGGATTGACAGATTGATAGCTC	CAAATCGCTCCACCAACTAAGAA
TGFBR2	GTAGCTCTGATGAGTGCAATGAC	CAGATATGGCAACTCCCAGTG
CD274 (PDL1)	TGGCATTTGCTGAACGCATTT	TGCAGCCAGGTCTAATTGTTTT
ENTPD1	AGGTGCCTATGGCTGGATTAC	CCAAAGCTCCAAAGGTTTCCT
LGALS1	TCGCCAGCAACCTGAATCTC	GCACGAAGCTCTTAGCGTCA
NT5E	GCCTGGGAGCTTACGATTTTG	TAGTGCCCTGGTACTGGTCG
PDCD1LG2 (PDL2)	CTCGTTCCACATACCTCAAGTCC	CTGGAACCTTTAGGATGTGAGTG
TGFB1	TCCTGGCGATACCTCAGCAA	GCCGGTAGTGAACCCGTTGAT
CD44	CTGCCGCTTTGCAGGTGTA	CATTGTGGGCAAGGTGCTATT
ANXA4	GGAGGTACTGTCAAAGCTGCT	GGCAAGGACGCTAATAATGGC
IL18	CAGATCGCTTCCTCTCGCAA	CCAGGTTTTCATCATCTTCAGCT AT
SNAI2	TGCGATGCCCAGTCTAGAAA	AGAAAAAGGCTTCTCCCCCGT
F11R	GTGCCTACTCGGGCTTTTCTT	GTCACCCGGTCCTCATAGGAA
PDLIM1	CCCAGCAGATAGACCTCCAG	TCTGAGCTTCCAAGTGTGTCATA

Supplemental Table S4. Source information for gene sets used in this study.

**Gene set accessions**		
Gene set	*No. of Genes*	*Source*
Smad2/3 Transcriptional Targets	843	GSEA-MSigDB; M2356
Verhaak_Mesenchymal	216	GSEA-MSigDB; M2122
Verhaak_Classical	161	GSEA-MSigDB; M2121
Verhaak_Proneural	177	GSEA-MSigDB; M2115
TREG vs TCONV	199	GSEA-MSigDB; M4650
TREG vs TEFF	200	GSEA-MSigDB; M3673
TREG vs TCONV_Activated	200	GSEA-MSigDB; M5690
Activated TREG vs Naïve CD4 TCELL	198	GSEA-MSigDB; M3550
Resting TREG vs Naïve CD4 TCELL	198	GSEA-MSigDB; M3542
TREG vs TCONV_TGFB-treated	199	GSEA-MSigDB; M5692
Treg (Regulatory T cell)	321	doi: 10.1016/j.humimm.2015.12.004
MDSC (Myeloid-Derived Suppressor Cell)	219	doi: 10.1126/sciimmunol.aay6017
M2 Macro (M2 Macrophage)	159	doi: 10.1038/s41598-020-73624-w
TAN (Tumor-Associated Neutrophil)	200	doi: 10.1038/s41590-022-01311-1
Pan-cancer Epithelial	25	doi: 10.1158/1078-0432.CCR-15-0876
Pan-cancer Mesenchymal	52	doi: 10.1158/1078-0432.CCR-15-0876

Supplemental Table S5. Source information for publicly available single-cell RNA-sequencing datasets used in this study.

**Publicly Available scRNA-seq Datasets**		
Tumor Type	*Depository & Accession*	*Publication*
Glioma	GEO; GSE182109	doi: 10.1038/s41467-022-28372-y
Renal Cell Carcinoma	TISCH2; ID: T020120; KIRC_GSE171306	doi: 10.1073/pnas.2103240118
Colorectal Cancer	TISCH2; ID: T020099; CRC_GSE166555	doi: 10.15252/emmm.202114123
Breast Cancer	TISCH2; ID: T010014; BRCA_GSE176078	doi: 10.1038/s41588-021-00911-1
Non-Small Cell Lung Cancer	TISCH2; ID: T020140; NSCLC_GSE148071	doi: 10.1038/s41467-021-22801-0
Pancreatic Ductal Adenocarcinoma	TISCH2; ID: T010066; PAAD_CRA001160	doi: 10.1038/s41422-019-0195-y
Melanoma Metastases	GEO; GSE185386	doi: 10.1016/j.cell.2022.06.007

Supplemental Table S6. Genes induced by Oct4 and Sox2 up-regulated in GBM. * = Genes significantly enriched in mesenchymal GBMs compared to classical and proneural.

TGFBR2*	COL5A2*	RRAS2	SNAI2*	PLS3	PCOLCE*
SPP1*	DPP4*	TGFBI*	FZD1*	PTGS1	ANXA4*
PROCR*	F11R*	RNASE4*	PRKCDBP*	FBN2	STC1*
CASP1*	DPYD*	GJA4*	TMEM45A	FAS*	FSTL1*
ANGPT1	RAB32*	SAP30L	STK17B*	RAB27A*	NDUFA4L2
PAPSS2*	MYL9*	SH3BP2	DDR2*	KIAA0040	SEC61G
F2RL1	SLC27A3	MTMR11	PLA2G5	SWAP70	EDNRB
ATP8B1	GPC3	RPL5	RPS8	SCUBE2	UST
SIX6	DPYSL3				

Supplementary Table S7. Treg-related genes induced by transgenic TGFBR2 in GSCs.

ABCC1	CEP55	FRMD4B	LAMC1	POFUT1	SCAMP1	SOX3	TNFSF10
ADAMTS6	CERK	FRRS1L	LAPTM4A	POLE	SDC4	SPAG17	TNFSF12
AGPAT4	CHORDC1	FTX	LINC00310	PPM1B	SEC24A	SPATA22	TOP3A
ALCAM	CHTF18	FUCA2	LNPK	PPP1R21	SEC24D	SPCS2	TOR1AIP1
ANGPTL1	CLDN12	FYN	LRIG1	PPP1R26-AS1	SELENOM	SPDYA	TP53
ANKRD13C	CLDND1	GALM	LRRC8D	PPP6R3	SEMA5A	SPIN4	TP53I13
ANKS1B	CLIC1	GATA1	LRRCC1	PRDM1	SEMA6A	SSR2	TPH1
ANXA2	COL1A2	GBP2	LYN	PRDM5	SEMA7A	STC2	TPR
ANXA2P2	COL7A1	GBP4	MAGI1	PRELID3B	SERPINC1	STIM2	TPRG1
ANXA4	CPD	GCA	MATN2	PRKRA	SETD6	STK32A	TRIM16
ANXA5	CPOX	GCNT1	MBNL3	PRNP	SF3B4	STON1	TRMO
APIP	CRTAP	GGH	MEGF9	PROS1	SGSH	SUCO	TRPC3
ARHGAP20	CSGALNACT2	GLB1	MEIS2	PSEN1	SH2D1A	SURF4	TSPAN6
ARL6IP1	CSRP2	GLCE	MGME1	PSRC1	SHTN1	SVIP	TTC12
ARMCX4	CTNS	GLIPR1L1	MIR22HG	PTBP2	SIPA1L2	SWAP70	TTC39C
ARMCX5	CXCL3	GNB5	MPRIP	PTPN7	SKI	TANK	TXNDC16
ATAD2	CYB561	GOLGA8B	MREG	PTPRJ	SKIL	TBK1	UBE2J1
ATG10	CYBRD1	GOSR1	MT1E	RAB11FIP2	SLC12A2	TBRG1	UBE4B
ATL3	DDX5	GPR19	MTERF1	RAB2B	SLC15A3	TCEAL8	UGGT1
ATP11A	DMD	GPR62	MYL9	RAD51B	SLC16A1	TCN2	USP11
ATP1B1	DNAH7	GPR85	MYO3B	RALB	SLC18B1	TDRD7	USP48
ATP2B4	DNAJC1	GSAP	NABP1	RANBP9	SLC22A1	TEAD1	UTRN
B2M	DNAJC12	GUCY1B1	NDFIP2	RANGRF	SLC25A24	TGFBR1	UTS2B
B4GALT4	DOK2	HDAC4	NFKBIZ	RBL1	SLC25A46	TGM2	VAMP3
B9D1	DYNLL1	HERPUD1	NR4A2	RBPMS	SLC27A2	THRB	VASN
BARD1	EBI3	HM13	NT5E	RDX	SLC35D1	TIFA	VEZT
BECN1	ECM1	HSPA1A	NUP155	REEP3	SLC39A7	TINF2	VPS54
BRCA1	EGR2	IBTK	OPTN	REEP5	SLC44A1	TIRAP	WDR33
BUB1B	ELL2	ICAM1	PCDH11X	RENBP	SLC44A3	TLCD1	WFS1
C1QTNF12	ELOVL5	IDS	PDCD1LG2	RHBDL2	SLC45A1	TLL2	WLS
C3orf80	ENTPD1	IER5	PDE8A	RHCG	SLC4A11	TLR4	WWP1
C6orf136	EPS15	IFT80	PHACTR2	RNF113A	SLC4A9	TLR7	YIPF2
CAPN5	ERBIN	IGF2R	PHIP	RNF167	SLC5A3	TMED3	YWHAE
CASP1	ERI1	IL10RB	PHKB	RNF19A	SLC8B1	TMEM109	ZC2HC1A
CASP3	ESS2	IL18	PHLDB2	ROBO1	SLC9A8	TMEM158	ZCCHC18
CASP8	F2RL2	IL2RB	PHTF2	ROS1	SMCHD1	TMEM208	ZCCHC8
CCDC50	FAAP24	IL7	PIAS3	RPE65	SNAI2	TMEM65	ZFAND5
CD200	FAM174A	IQGAP1	PIGX	RPL39L	SNHG12	TMEM70	ZFAND6
CD274	FAM174B	IQGAP3	PIK3AP1	RRM1	SNORA65	TMLHE	ZFYVE16
CD38	FAR1	ITGA6	PIP5K1B	RUBCNL	SNRNP40	TMTC3	ZIC1
CD58	FCHO2	ITGAE	PIWIL4	RUNX2	SNX16	TNF	ZNF131
CD81	FES	JADE1	PLAGL1	S100A16	SNX3	TNFAIP3	ZNF430
CD83	FGL2	KCNMB4	PLP2	S100A4	SOCS2	TNFRSF18	ZNF432
CDHR3	FHL2	KIF5C	PLS3	SAMD8	SOD2	TNFRSF1B	ZNF644
CDKN2B	FMNL2	KLHL2	PLXND1	SAR1B	SOX2-OT	TNFRSF9	ZSCAN26
CEP162	FNBP1L						
